# Prevalence of Burnout in Medical and Surgical Residents: A Meta-Analysis

**DOI:** 10.3390/ijerph16091479

**Published:** 2019-04-26

**Authors:** Zhi Xuan Low, Keith A. Yeo, Vijay K. Sharma, Gilberto K. Leung, Roger S. McIntyre, Anthony Guerrero, Brett Lu, Chun Chiang Sin Fai Lam, Bach X. Tran, Long H. Nguyen, Cyrus S. Ho, Wilson W. Tam, Roger C. Ho

**Affiliations:** 1 Department of Psychological Medicine, Yong Loo Lin School of Medicine, National University of Singapore, Singapore 119228, Singapore; a0133292@u.nus.edu (Z.X.L.); a0133415@u.nus.edu (K.A.Y.); 2 Department of Medicine, Yong Loo Lin School of Medicine, National University of Singapore, Singapore 119228, Singapore; mdcvks@nus.edu.sg; 3 Department of Surgery, The University of Hong Kong, Hong Kong, China; gilberto@hku.hk; 4 Institute of Medical Science, University of Toronto, Toronto, ON M5S 1A8, Canada; Roger.McIntyre@uhn.ca; 5 Mood Disorders Psychopharmacology Unit, University Health Network, Toronto, ON M5G 2C4, Canada; 6 Department of Psychiatry, University of Toronto, Toronto, ON M5T 1R8, Canada; 7 Department of Toxicology and Pharmacology, University of Toronto, Toronto, ON M5S 1A8, Canada; 8 Department of Psychiatry, John A Burns School of Medicine, University of Hawaii, Honolulu, HI 96813, USA; GuerreroA@dop.hawaii.edu (A.G.); brettlu@yahoo.com (B.L.); 9 South London and Maudsley NHS Foundation Trust, London SE5 8AZ, UK; sinfailamcc@googlemail.com; 10 Institute for Preventive Medicine and Public Health, Hanoi Medical University, Hanoi 100000, Vietnam; bach@jhu.edu; 11 Department of Health, Behavior and Society, Johns Hopkins Bloomberg School of Public Health, Baltimore, MD 21205, USA; 12 Vietnam Young Physicians’ Association, Hanoi 100000, Vietnam; 13 Institute for Global Health Innovations, Duy Tan University, Da Nang 550000, Vietnam; long.ighi@gmail.com; 14 Department of Psychological Medicine, National University Hospital, Singapore 119228, Singapore; su_hui_ho@nuhs.edu.sg; 15 Alice Lee School of Nursing, Yong Loo Lin School of Medicine, National University of Singapore, Singapore 117597, Singapore; nurtwsw@nus.edu.sg; 16 Biomedical Institute for Global Health Research and Technology, National University of Singapore, Singapore 119228, Singapore; 17 Centre of Excellence in Behavioral Medicine, Nguyen Tat Thanh University (NTTU), Ho Chi Minh City 70000, Vietnam

**Keywords:** burnout, junior doctors, medical, meta-analysis, prevalence, residency, surgical

## Abstract

The burnout syndrome is characterized by emotional exhaustion, depersonalization, and reduced personal achievement. Uncertainty exists about the prevalence of burnout among medical and surgical residents. Associations between burnout and gender, age, specialty, and geographical location of training are unclear. In this meta-analysis, we aimed to quantitatively summarize the global prevalence rates of burnout among residents, by specialty and its contributing factors. We searched PubMed, PsycINFO, Embase, and Web of Science to identify studies that examined the prevalence of burnout among residents from various specialties and countries. The primary outcome assessed was the aggregate prevalence of burnout among all residents. The random effects model was used to calculate the aggregate prevalence, and heterogeneity was assessed by I^2^ statistic and Cochran’s Q statistic. We also performed meta-regression and subgroup analysis. The aggregate prevalence of burnout was 51.0% (95% CI: 45.0–57.0%, I^2^ = 97%) in 22,778 residents. Meta-regression found that the mean age (β = 0.34, 95% CI: 0.28–0.40, *p* < 0.001) and the proportion of males (β = 0.4, 95% CI = 0.10–0.69, *p* = 0.009) were significant moderators. Subgroup analysis by specialty showed that radiology (77.16%, 95% CI: 5.99–99.45), neurology (71.93%, 95% CI: 65.78–77.39), and general surgery (58.39%, 95% CI: 45.72–70.04) were the top three specialties with the highest prevalence of burnout. In contrast, psychiatry (42.05%, 95% CI: 33.09–51.58), oncology (38.36%, 95% CI: 32.69–44.37), and family medicine (35.97%, 95% CI: 13.89–66.18) had the lowest prevalence of burnout. Subgroup analysis also found that the prevalence of burnout in several Asian countries was 57.18% (95% CI: 45.8–67.85); in several European countries it was 27.72% (95% CI: 17.4–41.11) and in North America it was 51.64% (46.96–56.28). Our findings suggest a high prevalence of burnout among medical and surgical residents. Older and male residents suffered more than their respective counterparts.

## 1. Introduction

The term “burnout” was first defined by Freudenberger to describe the emotional exhaustion experienced by civil servants [1]. The three main components of burnout are an overwhelming exhaustion, feelings of cynicism or depersonalization, and a sense of ineffectiveness and lower efficacy [2,3,4]. The processual character of burnout refers to cumulative negative consequences of long-term work-related stress as a result of exhaustion [5]. In clinical settings, evidence shows that burnout causes prescription errors [6] and reduces the quality of medical services [7,8,9], potentially affecting inter-professional relationship [10,11]. Burnout is a precipitating factor for depression [12] and substance abuse [13] among medical professionals. It is also one of the most common mental health issues faced by medical and surgical residents or trainees who are junior doctors holding Bachelor of Medicine and Bachelor of Surgery or undergraduate Doctor of Medicine degrees and who are undergoing supervised medical or surgical specialty training. Burnout contributes to poor job satisfaction [14] and a negative impact on their mental and physical health [15,16]. In recent years, the risk of residents developing burnout has been further aggravated by increasing peer competition, clinical and administrative loads, medical litigation, and expectations of training [17]. However, this is often overlooked by clinical supervisors and hospital administration.

Although burnout among residents remains under-recognized, many important issues are open for debate. First, the global prevalence of burnout in residents is unclear due to different working conditions in various medical and surgical specialties. Previous reviews focused on the prevalence rates of burnout syndrome in a single specialty [18,19]. Second, variations have been reported in the prevalence rates of burnout, ranging from 18.7% to 74.8%, mainly due to different assessment methods and small sample sizes [20]. Third, the influence of demographic variables (e.g., sex, age, geographic region) on burnout rates has not been adequately tested. As a result, there is a strong justification for conducting research on the prevalence of burnout. In this meta-analysis, we aimed to synthesize data on the prevalence of burnout in residents from various specialties in different world regions. Furthermore, in this study, we evaluated the demographic and geographical moderators which might facilitate better identification of residents at risk and guide health authorities when they plan optimal preventive interventions.

## 2. Materials and Methods

### 2.1. Search Strategy and Selection Criteria

The literature search and the review protocol were designed and performed in accordance with the Preferred Reporting Items for Systematic Reviews and Meta-Analyses (PRISMA) statement [21]. A systematic search using PubMed, PsycINFO, Embase, and Web of Science was independently conducted by two authors (Z.X.L. and K.A.Y.), from inception to March 2018. The search was done with Boolean operators “AND” and “OR”, and with all possible combinations of the Medical Subject Heading (MeSH) terms: residents, trainees, burnout, burn-out, burn out, medical, medicine, internal medicine, general surgery, surgery, surgical, radiology, radiological, neurology, neurological, orthopaedics, orthopedics, orthopaedic, orthopedic, dermatology, obstetrics, obstetric, gynecology, gynaecology, gynecological, gynaecological, neurosurgery, neurosurgical, paediatrics, pediatrics, paediatric, pediatric, anaesthesia, anesthesia, anaesthesiology, anesthesiology, otolaryngology, ear nose and throat, ENT, psychiatry, psychiatric, oncology, oncological, family medicine, emergency medicine, accident and emergency, and ophthalmology and ophthalmological. Back-referencing was used to identify potential studies and relevant citations to be included in our analysis.

### 2.2. Inclusion and Exclusion Criteria

Study inclusion criteria were as follows: observational cohort and cross-sectional studies that reported the prevalence of burnout among medical residents and the measurement of burnout using the Maslach Burnout Inventory (MBI) [22]. *Burnout* is a *long*-*term stress reaction marked by emotional exhaustion*, *depersonalization, and a lack of sense of personal accomplishment* [23]. Medical/surgical residents or trainees were defined as junior doctors who possessed basic medical degrees such as Bachelor of Medicine (MB), Bachelor of Medicine and Surgery (MBBS, MBChB, or equivalent), or Doctor of Medicine (MD) and who were undergoing supervised training. Other professionals such as senior physicians, chiropractors, and medical students were excluded. We also excluded studies that had missing or unavailable data, such as the specialty being studied, the number of residents who experienced burnout, or the prevalence of burnout. Finally, systematic reviews, commentaries, editorial articles, and publications that were not written in English were also excluded.

### 2.3. Data Extraction and Quality Assessment

We used a standard data collection form to record study characteristics, participant demographics, and various results. The primary outcome of our meta-analysis was the prevalence of burnout in a particular group of residents being studied. Two co-authors (Z.X.L. and K.A.Y.) independently extracted the data. Disagreements were resolved by discussion with the last author (R.C.H.).

The methodological quality assessment of the included studies was performed with the National Institutes of Health’s Quality Assessment Tool for Observational Cohort and Cross-Sectional Studies (NIH-QAT) [24] (See Appendix A). This tool helps researchers to assess various aspects of a study and assign an overall quality rating of “Good”, “Fair”, or “Poor”. The assessment criteria include clarity of the research question, consistency of study population and eligibility criteria, justification of sample size, outcome measurement; duration of outcome measurement and follow-ups, and quality of statistical analyses.

### 2.4. Statistical Analyses

Statistical analyses were conducted with the “metafor” function of the software R (R Core Team, Vienna, Austria, 2013). We calculated prevalence rates of burnout based on the crude numerators (i.e., the number of residents who met burnout criteria) and denominators (i.e., total number of residents) provided by individual studies. The random effects model generalizes findings beyond the included studies by assuming that the selected studies are random samples from a larger population [25]. A random effects model was used to calculate the aggregate prevalence of burnout and 95% confidence intervals (CIs) [26]. Heterogeneity was examined by Cohen’s Q statistic and I^2^ statistic [27]. As a guide, I^2^ values of 25% may be considered low, 50% moderate, and 75% high [28]. In the presence of high heterogeneity, we used the random effects model which is deemed most appropriate [29]. Furthermore, we performed meta-regression to assess the influence of different study factors on the aggregate prevalence of burnout [30]. Egger’s regression test was performed to assess for the presence of publication bias [31]. Subgroup analyses were conducted to explore the source of heterogeneity among subgroups—by specialty and geographical region.

## 3. Results

### 3.1. Characteristics of Studies

**Figure 1:**
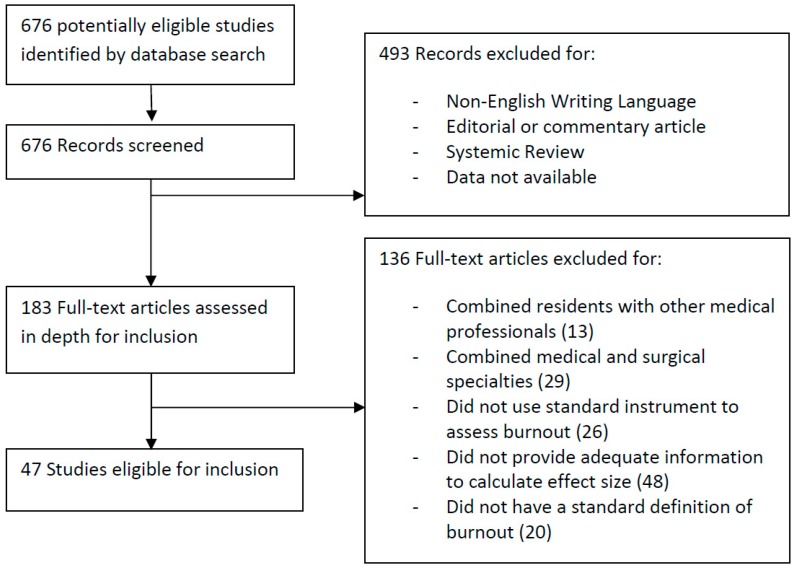
Study selection process.

Our search strategy identified 676 potentially eligible studies. We screened the titles and abstracts and excluded 493 irrelevant articles due to various pre-defined criteria. The full texts of the remaining 183 articles were assessed for eligibility, of which 136 were excluded (Figure 1). Finally, 47 articles were included in the meta-analysis.

Table 1 summarizes the characteristics of the included studies. For this study, thirty-seven (37) (78.72%) studies were from North and South America (Canada, USA, Brazil) [32,33,34,35,36,37,38,39,40,41,42,43,44,45,46,47,48,49,50,51,52,53,54,55,56,57,58,59,60,61,62,63,64,65,66], three (3) studies (6.38%) were from Europe (France, Spain) [67,68,69], five (5) studies (10.63%) were from Asia (Pakistan, Saudi Arabia, Turkey) [70,71,72,73,74], as well as one (1) study (2.13%) each from Africa (Egypt) [75] and Australia [76]. The mean age of individual participants varied from 25.9 to 32.0 years, with the proportion of male residents ranging from 10% to 88%.

### 3.2. Aggregate Prevalence of Burnout

**Figure 2:**
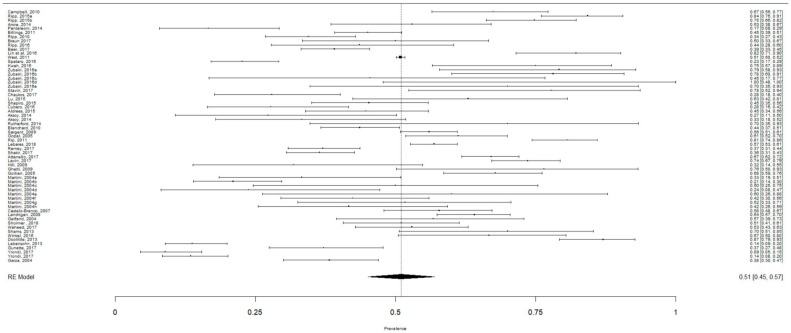
The aggregate prevalence of burnout in all residents.

A total of 22,778 individual participants were included in the meta-analysis to calculate the aggregate prevalence of burnout (Figure 2). The aggregate prevalence of burnout was 51.0% (95% CI: 45.0–57.0%, I^2^ = 96.96%). Publication bias was not present as confirmed by the Egger’s regression test (intercept = −0.051, *p* = 0.95). Meta-regression found that the mean age of residents (β = 0.34, 95% CI: 0.28–0.40, *p* < 0.001) and proportion of males (β = 0.4, 95% CI = 0.1–0.69, *p* = 0.009) were significant moderators. The publication year of study (β = −0.0036, 95% CI: −0.014–0.0071, *p* = 0.51) and response rate of residents (β = −0.086, 95% CI: −0.24–0.072, *p* = 0.28) were not statistically significant moderators.

**Figure 3:**
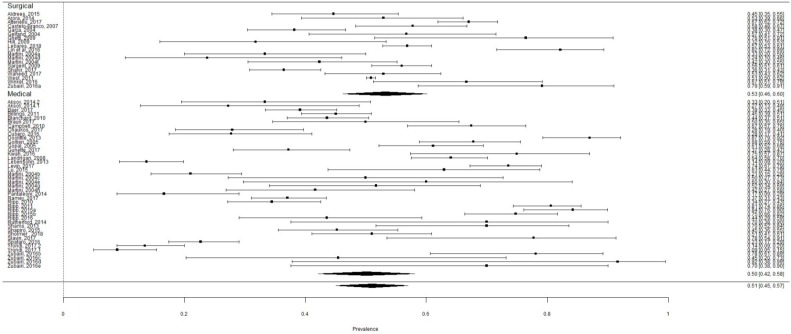
The aggregate prevalence of burnout in medical and surgical residents.

There were 18,759 (82.36%) residents in surgical residencies including general surgery, neurosurgery, obstetrics and gynecology, ophthalmology, orthopedics, and otolaryngology. The prevalence rate of burnout in surgical residents was 53.27% (95% CI: 46.27–60.15%) (Figure 3). There were 4019 (17.64%) residents in medical residencies including anesthesia, dermatology, emergency medicine, family medicine, internal medicine, neurology, oncology, pediatrics, psychiatry, and radiology. The prevalence rate of burnout in medical residents was 50.13% (95% CI: 42.12–58.13%) (Figure 3). Although the prevalence of burnout was higher among surgical residents, the difference was not statistically significant (Q = 0.92, *p* = 0.34).

### 3.3. Subgroup Analysis

In the subgroup analysis by specialty (Table 2), radiology (77.16%, 95% CI: 5.99–99.45), neurology (71.93%, 95% CI: 65.78–77.39), and general surgery (58.39%, 95% CI: 45.72–70.04) were the top three specialties with the highest prevalence rates of burnout. In addition, more than 50% of residents experienced burnout in internal medicine (57.11%, 95% CI: 45.11–68.33), orthopedics (55.63%, 95% CI: 50.93–60.28), dermatology (51.89%, 95% CI: 42.42–61.21), obstetrics and gynecology (52.84%, 95% CI: 41.77–63.63), and neurosurgery (52.02%, 95% CI: 31.02–72.33). In contrast, psychiatry (42.05%, 95% CI: 33.09–51.58), oncology (38.36%, 95% CI: 32.69–44.37), and family medicine (35.97%, 95% CI: 13.89–66.18) had the lowest prevalence rates of burnout. However, there was no statistically significant difference in prevalence rates among various specialties (Q = 13.9, *p* = 0.53). In the subgroup analysis by geographical region (Table 2), several European countries had the prevalence of burnout 27.72% (95% CI: 17.4–41.11). Several Asian countries had the highest prevalence of burnout 57.18% (95% CI: 45.8–67.85). However, the difference in prevalence rates among various continents was not statistically significant (Q = 9.43, *p* = 0.093).

## 4. Discussion

This meta-analysis compared burnout prevalence rates by medical specialty, and summarized data on the overall prevalence of burnout in residents. There were several important findings. First, the global prevalence of burnout among residents was considerably high—over 50%. Second, the prevalence of burnout was comparable between medical and surgical residents. Third, more than half of the residents from eight specialties (radiology, neurology, general surgery, internal medicine, orthopedics, dermatology, obstetrics and gynecology, and neurosurgery) reported burnout, although there was no statistically significant difference in prevalence rates among various specialties. Older age and male gender were associated with higher prevalence of burnout. Interestingly, the year of publication was not a significant moderator, suggesting that the prevalence of burnout does not change with time.

### 4.1. Potential Reasons for High Prevalence of Burnout in Some Medical and Surgical Specialities

We found that more than half of medical and surgical residents experienced burnout. Symptoms of burnout can originate from many causes, such as bureaucratic requirements [78], continually changing work environments [17], micro-management by the administration, poor clinical supervision, sensationalist media reports of medical errors [79], limited healthcare resources [17], litigious environments [80], and poor work–life balance [81]. The prevalence of burnout among psychiatry residents was less than 50%. This finding is not surprising because psychiatry residency offers training in different modalities of psychotherapy, including cognitive behavior therapy, interpersonal therapy, supportive psychotherapy, and problem-solving psychotherapy. Perhaps, psychiatry residents can apply psychotherapeutic techniques to reduce or overcome symptoms of burnout and negative emotion. In contrast, we found that radiology residents had the highest prevalence rate of burnout. The radiology training lacks direct interaction with patients and focuses on the technical aspects of various imaging modalities and interpretation of images to establish diagnoses. As a result, the radiology residents may lack clinical and psychotherapeutic skills to handle burnout and negative emotion. Furthermore, the radiology residents work in academic institutions, where they are often worried about errors in diagnosis and criticism by other specialties. The insecurity may increase further with the advent of artificial intelligence in the interpretation of images, which could impact a sense of potential job displacement [82].

### 4.2. Extension of Burnout from Medical Schools into Residencies

The present study helps illuminate some of the factors that were not apparent in previous studies of burnout among medical students and trainees. A recent meta-analysis reported that burnout ranged from 7% to 75% among medical students [83]. However, this study did not provide an aggregate prevalence of burnout or examine possible geographical differences. Nevertheless, burnout in residency could originate from burnout in medical school, possibly due to a lack of awareness and intervention by universities and health authorities. Burnout and depression are interrelated [84]. Medical students with burnout are more likely to experience depression, which is associated with work-related disability and loss of productivity after graduation [85]. Puthran et al. (2016) [31] performed a meta-analysis and found that the global prevalence of depression among medical students was 28%. Graduate medical program (e.g., the M.D. system in North America) and receiving medical education in Middle Eastern countries were factors contributing to depression among medical students. Similarly, older age was a significant moderator contributing to burnout among residents. Lastly, residents are finding themselves working in roles for which they were not trained in the medical schools, including hospital accreditation processes, clinical audits, and administrative and teaching roles, all of which could contribute to burnout in residency.

### 4.3. Comparison with Other Healthcare Professions

Similar to medical students, other healthcare professionals also suffer from burnout. Monsalve-Reyes et al. (2018) reported a relatively lower (31%) prevalence of burnout among 1110 primary care nurses [86]. In our meta-analysis, family medicine residents had the lowest aggregate prevalence of burnout among all residents, which is different from a study conducted in the USA [87]. Similar to family medicine residents, primary care nurses manage stable patients with chronic diseases in their homes and communities, which allows them to form stronger bonds with the patients and helps prevent burnout [86]. In contrast, residents from hospital-based specialties often manage unstable patients with acute diseases with various complications, which may contribute to a higher prevalence of burnout. Furthermore, residents who receive hospital-based training are more likely to face litigations, compared with residents in primary care [17].

We found high between-study heterogeneity (>90%) among neurosurgery, orthopedics, internal medicine, general surgery, anesthesia, and pediatrics. This might be due to different countries and their residency structures, assessment methods, medico-legal practices, number of stay-in calls, pay scales, workplace conditions, job security, and employment opportunities.

### 4.4. Differences in Residency Burnout between the East and West

Some of these observations are substantiated by our subgroup analyses that showed differences in the prevalence rates of burnout among different continents, although such differences were not statistically significant. The contributing factors to high prevalence of burnout among residents in some Asian countries could include long working hours, high educational pressures, lack of autonomy, high levels of work–home intrusion, and professional uncertainty [88,89]. These stressors are prevalent in some Asian countries. Furthermore, there is no advocacy to safeguard working conditions for residents in some Asian countries. In contrast, the European Working Time Directive stated a maximum of 48 working hours per week, and residency training programs in European countries demonstrated good compliance [90]. Interestingly, the prevalence of burnout in North America was higher than in several European countries. That might be explained by differences in work hours and compliance rates. The Accreditation Council for Graduate Medical Education of the USA allows an 80-h work limit per week for residents [91], and the compliance rates of residency programs were low [92].

### 4.5. Future Research

We found that older age of residents was significantly associated with higher prevalence of burnout. There are several postulations. First, older residents might enter residency late due to difficulty in choosing the specialty. Second, older residents might find it difficult to cope with the training demands and postgraduate examinations as compared with younger residents. Third, older residents are more likely to be married and could be coping with family commitments as well as the need to sit for examinations, conduct research, and perform administrative duties [93]. This observation might explain why male gender, especially married male residents, was significantly associated with higher prevalence of burnout. This is an important finding since men are less likely to admit psychological suffering and seek help as compared with women [94].

Future research should include a prospective study to evaluate the psychological, occupational, training, and sociodemographic factors that may influence the development of burnout syndrome among residents. None of the studies included in this meta-analysis assessed associations between burnout and employment rates of residents. Future research should examine this and other long-term effects of burnout. Residents often deal with burnout by avoidance and denial [95]. Neglected burnout often leads to alcohol and substance misuse, anxiety, depression, discontinuation of residency, fatigue, impaired interpersonal and marital relationship, and insomnia [17]. It is unclear why mental health has been neglected as part of the occupational health agenda for medical and surgical residents. It may be that training administrators are unaware of burnout, or that they believe current practices help strengthen medical practitioners for real-world working conditions.

### 4.6. Policy Implications

Our findings have important policy implications. Policymakers should prepare health systems for the proper management of burnout in residents, including evidence-based psychological interventions. Health authorities should offer early detection and prevention programs to tackle burnout in residents. Specialties with very high burnout rates (>50%) should consider structural and organizational changes in the training program to improve the training environment, competency of trainers, opportunities for career development, and satisfaction of residents. Residency programs in some Asian countries, and elsewhere, should improve work–life balance and set limits on working hours.

### 4.7. Strengths and Limitations

Strengths of this meta-analysis include the use of a comprehensive search strategy, the involvement of at least two independent researchers throughout the research process. Due to the robust search strategy, the sample size of our meta-analysis (*n* = 22,778) was almost five times higher than a recent meta-analysis on burnout of medical residents (*n* = 4664) [96]. This meta-analysis included studies which used the Maslach Burnout Inventory (MBI). The lack of publication bias suggests that we were unlikely to miss studies that could have altered the results of our meta-analysis. Our approach avoided different ways to define and measure burnout, which is the main limitation of current burnout research [97]. Unless burnout can be measured by neuroimaging or other biological methods as in some psychiatric symptoms [98,99], the MBI remains the most established way to measure burnout when this meta-analysis was conducted.

This meta-analysis has several limitations. First, all included studies were observational which could lead to inherent bias because of unmeasured confounders, including workloads and resources for each residency program, genetic risk for depression, past psychiatric illness, and substance abuse. Similarly, we were unable to extract some correlates of burnout (e.g., weekly working hours, marital status, financial status, job satisfaction) as the underlying data sets did not provide such information. Second, although mean age and proportion of males were moderators for meta-regression, it is important to note that 38 out of 60 studies (63.33%) and 21 out of 60 studies (35%) did not report mean age and proportion of gender, respectively. Furthermore, the results of meta-regressions suggest observational associations but not causations due to ecological fallacy [100]. Third, this study was aimed at evaluating the prevalence of burnout among residents using the largest number of studies possible, across all specialties, but the distribution of the number of residents per specialty was uneven. This meta-analysis included 22,778 residents, of whom 17,153 belonged to surgery, with the next highest group belonging to internal medicine at 4019, whereas other specialties had much lower residents with psychiatry at 245, neurology at 228, family medicine at 213, and radiology at 99. This is one of the key limitations of this meta-analysis as some of the specialties were under-represented. Fourth, we were not able to compare the prevalence of individual components of burnout (e.g., low personal achievement), due to differences in how studies assessed burnout symptoms. Lastly, many of the studies were conducted in the USA, with fewer conducted in developing countries (e.g., Vietnam) and emerging economies (e.g., China, India) where residents encounter heavy workloads, low wages, and lack of respect from patients [101,102]. Of particular note, there is a paucity of research in Europe, Asia, Australia, and Africa and on some specialties including emergency medicine and ophthalmology. As a result, the subgroup analysis based on continents should be interpreted with caution.

## 5. Conclusions

The results of this meta-analysis suggest a high prevalence of burnout in residents—over 50%. Our findings showed that burnout is prevalent in all specialties, but that some specialties have much higher rates than others. Results also demonstrated that age, sex, and geographic location could all influence burnout rates. Burnout has negative impacts on job satisfaction, the health of the residents, and the delivery of clinical services to patients. More studies are required to identify plausible causal pathways between residency training and burnout. Policymakers and health authorities should use available evidence to help immediately improve detection, overall management, and prevention of burnout in residents. Our findings suggest the urgent need for structural and organizational changes for residency programs, specific to local training environment and other demographic factors, to reduce the prevalence of burnout among residents.

## Figures and Tables

**Table 1:** Characteristics of included studies.

Study	Study Demographics				Study Results					MBI/Abbrev. MBI
	Country	Region	Mean Age	Proportion of Males	Specialty	Medical/Surgical	Response Rate	Sample Size	Number of Residents Who Reported Burnout	
Garza et al., 2004 [45]	USA	N. America	NR	0.29	Obstetrics and Gynecology	Surgical	37%	136	52	MBI
Gelfand et al., 2004 [46]	USA	N. America	NR	NR	General Surgery	Surgical	69%	37	21	MBI
Martini et al., 2004a [44]	USA	N. America	NR	NR	Obstetrics and Gynecology	Surgical	35%	36	12	MBI
Martini et al., 2004b [44]	USA	N. America	NR	NR	Internal Medicine	Medical	35%	114	24	MBI
Martini et al., 2004c [44]	USA	N. America	NR	NR	Neurology	Medical	35%	16	8	MBI
Martini et al., 2004d [44]	USA	N. America	NR	NR	Ophthalmology	Surgical	35%	21	5	MBI
Martini et al., 2004e [44]	USA	N. America	NR	NR	Dermatology	Medical	35%	10	6	MBI
Martini et al., 2004f [44]	USA	N. America	NR	NR	General Surgery	Surgical	35%	59	25	MBI
Martini et al., 2004g [44]	USA	N. America	NR	NR	Psychiatry	Medical	35%	29	15	MBI
Martini et al., 2004h [44]	USA	N. America	NR	NR	Family Medicine	Medical	35%	36	15	MBI
Goitein et al., 2005 [43]	USA	N. America	NR	0.47	Internal Medicine	Medical	73%	118	80	MBI
Gopal et al., 2005 [42]	USA	N. America	29.9	0.58	Internal Medicine	Medical	87%	121	74	MBI
Castelo-Branco et al., 2007 [68]	Spain	Europe	27.0	0.14	Obstetrics and Gynecology	Surgical	67%	109	63	MBI
Landrigan et al., 2008 [41]	USA	N. America	30.2	0.29	Pediatrics	Medical	59%	220	141	MBI
Ghetti et al., 2009 [40]	USA	N. America	28.0	NR	Obstetrics and Gynecology	Surgical	47%	17	13	MBI
Hill and Smith, 2009 [48]	USA	N. America	NR	NR	Otolaryngology	Surgical	76%	22	7	MBI
Sargent et al., 2009 [39]	USA	N. America	NR	0.88	Orthopedics	Surgical	NR	384	215	MBI
Blanchard et al., 2010 [67]	France	Europe	28.0	0.40	Oncology	Medical	60%	204	89	MBI
Campbell et al., 2010 [47]	USA	N. America	30.0	0.51	Internal Medicine	Medical	48%	86	58	MBI
Ripp et al., 2010 [38]	USA	N. America	NR	0.50	Internal Medicine	Medical	94%	145	50	MBI
Billings et al., 2011 [37]	USA	N. America	NR	NR	Internal Medicine	Medical	43%	284	128	Abbrev. MBI
Ripp et al., 2011 [20]	USA	N. America	NR	0.48	Internal Medicine	Surgical	73%	191	154	MBI
West et al., 2011 [36]	USA	N. America	NR	0.57	General Surgery	Surgical	77%	16,394	8343	Abbrev. MBI
Doolittle et al., 2013 [35]	USA	N. America	30.0	0.50	Internal Medicine	Medical	63%	108	94	MBI
Lebensohn et al., 2013 [34]	USA	N. America	29.0	0.40	Family Medicine	Medical	77%	167	23	MBI
Shams and El-Masry, 2013 [75]	Egypt	Africa	NR	NR	Anesthesia	Medical	73%	30	21	MBI
Aksoy et al., 2014a [70]	Turkey	Asia	25.9	0.45	Pediatrics	Medical	66%	22	6	MBI
Aksoy et al., 2014b [70]	Turkey	Asia	26.6	0.48	Internal Medicine	Medical	66%	33	11	MBI
Arora et al., 2014 [76]	Australia	Oceania	NR	0.88	Orthopedics	Surgical	22%	51	27	MBI
Pantaleoni et al., 2014 [33]	USA	N. America	NR	NR	Pediatrics	Medical	100%	54	9	MBI
Rutherford and Oda, 2014 [65]	Canada	N. America	29.5	0.10	Family Medicine	Medical	4%	10	7	MBI
Aldrees et al., 2015 [71]	Saudi Arabia	Asia	29.0	0.67	Otolaryngology	Surgical	69%	85	38	MBI
Lu et al., 2015 [32]	USA	N. America	NR	NR	Emergency Medicine	Medical	50%	27	17	MBI
Shapiro et al., 2015 [58]	USA	N. America	NR	0.51	Internal Medicine	Medical	77%	95	43	MBI
Ripp et al., 2015a [63]	USA	N. America	NR	0.44	Internal Medicine	Medical	62%	108	91	MBI
Ripp et al., 2015b [63]	USA	N. America	NR	0.58	Internal Medicine	Medical	71%	123	92	MBI
Cubero et al., 2016 [77]	Brazil	S. America	28.4	0.54	Oncology	Medical	31%	54	15	MBI
Lin et al., 2016 [62]	USA	N. America	30.8	0.58	General Surgery	Surgical	63%	73	60	MBI
Spataro et al., 2016 [61]	USA	N. America	29.9	0.51	Internal Medicine	Medical	69%	198	45	MBI
Kwah et al., 2016 [73]	Pakistan	Asia	NR	NR	Internal Medicine	Medical	59%	32	24	MBI
Ripp et al., 2016 [60]	USA	N. America	NR	NR	Internal Medicine	Medical	76%	39	17	MBI
Winkel et al., 2016 [59]	USA	N. America	NR	NR	Obstetrics and Gynecology	Surgical	64%	42	28	MBI
Zubairi and Noordin, 2016a [72]	Pakistan	Asia	NR	0.54	General Surgery	Surgical	54%	24	19	MBI
Zubairi and Noordin, 2016b [72]	Pakistan	Asia	NR	0.54	Internal Medicine	Medical	54%	32	25	MBI
Zubairi and Noordin, 2016c [72]	Pakistan	Asia	NR	0.54	Pediatrics	Medical	54%	11	5	MBI
Zubairi and Noordin, 2016d [72]	Pakistan	Asia	NR	0.54	Radiology	Medical	54%	5	5	MBI
Zubairi and Noordin, 2016e [72]	Pakistan	Asia	NR	0.54	Anesthesia	Medical	54%	10	7	MBI
Attenello et al., 2017 [66]	USA	N. America	30.9	0.78	Neurosurgery	Surgical	21%	346	232	Abbrev. MBI
Baer et al., 2017 [57]	USA	N. America	29.4	0.21	Pediatrics	Medical	53%	258	101	Abbrev. MBI
Braun et al. 2017 [64]	USA	N. America	28.6	0.79	Internal Medicine	Medical	30%	38	19	MBI
Busis et al., 2017 [54]	USA	N. America	32.0	0.51	Neurology	Medical	38%	212	156	MBI
Chaukos et al., 2017 [56]	USA	N. America	28.3	0.40	Psychiatry	Medical	80%	68	19	MBI
Guenette and Smith, 2017 [55]	USA	N. America	NR	0.63	Radiology	Medical	20%	94	35	MBI
Ramey et al., 2017 [53]	USA	N. America	NR	0.69	Oncology	Medical	32%	232	86	MBI
Shakir et al., 2017 [52]	USA	N. America	NR	0.80	Neurosurgery	Surgical	21%	255	93	Abbrev. MBI
Slavin et al., 2017 [51]	USA	N. America	NR	NR	Pediatrics	Medical	NR	18	14	MBI
Waheed et al., 2017 [74]	Pakistan	Asia	27.5	NR	Obstetrics and Gynecology	Surgical	NR	102	54	MBI
Yrondi et al., 2017a [69]	France	Europe	28.8	0.55	Anesthesia	Medical	NR	123	11	MBI
Yrondi et al., 2017b [69]	France	Europe	27.7	0.33	Psychiatry	Medical	NR	148	20	MBI
Lebares et al., 2018 [50]	USA	N. America	NR	0.49	General Surgery	Surgical	10%	566	322	MBI
Shoimer et al., 2018 [49]	Canada	N. America	NR	NR	Dermatology	Medical	59%	96	49	MBI

Notes: Papers which analyzed more than one cohort of residencies (for example, residents from different batches or specialties) are given specific letters of the alphabet (a, b, c, etc.) in their suffixes. Abbreviations: MBI = Maslach Burnout Inventory, Abbrev. MBI = Abbreviated version of MBI, N. America = North America, S. America = South America, NR = Not reported.

**Table 2:** Prevalence of burnout in residents by subgroup analysis.

Medical Specialty/Region	Number of Residents (pct.)	Burnout Prevalence pct. and 95% CI	I^2^
All residents	22,778 (100%)	51.0% (45.0–57.0)	97.0%
Surgical vs. medical: *p* (subgroup difference) = 0.337			
Surgical residents	18,759 (82.36%)	53.27% (46.27–60.15)	94.8%
Medical residents	4019 (17.64%)	50.13% (42.12–58.13)	95.0%
* Specialty: *p* (subgroup difference) = 0.533			
Radiology	99 (0.43%)	77.16% (5.99–99.45)	77.8%
Neurology	228 (1%)	71.93% (65.78–77.39)	0%
General Surgery	17,153 (75.31%)	58.39% (45.72–70.04)	96.0%
Internal Medicine	1865 (8.19%)	57.11% (45.11–68.33)	95.3%
Orthopedics	435 (1.91%)	55.63% (50.93–60.28)	96.3%
Dermatology	106 (0.47%)	51.89% (42.42–61.21)	0%
Obstetrics and Gynecology	442 (1.94%)	52.84% (41.77–63.63)	78.0%
Neurosurgery	601 (2.63%)	52.02% (31.02–72.33)	96.3%
Pediatrics	583 (2.6%)	43.74% (26.70–62.39)	92.3%
Anesthesia	163 (0.71%)	43.71% (11.15–82.29)	92.3%
Otolaryngology	107 (0.47%)	42.06% (33.09–51.58)	0.0%
Psychiatry	245 (1.08%)	42.05% (33.09–51.58)	89.6%
Oncology	490 (2.15%)	38.36% (32.69–44.37)	27.6%
Family Medicine	213 (0.94%)	35.97% (13.89–66.18)	88.4%
^†^ Region: *p* (subgroup difference) = 0.093			
Several Asian countries (Pakistan, Saudi Arabia, and Turkey)	356 (1.56%)	57.18% (45.80–67.85)	80.9%
Several European countries (France, Spain)	584 (2.56%)	27.72% (17.40–41.11)	96.4%
North America	21,757(95.52%)	51.64% (46.96–56.28)	97.1%

* Aggregate prevalence rates of burnout in emergency medicine and ophthalmology were not included due to inadequate number of studies. ^†^ Aggregate prevalence rates of burnout in Africa and Oceania were not included due to inadequate number of studies.

**Figure A1:**
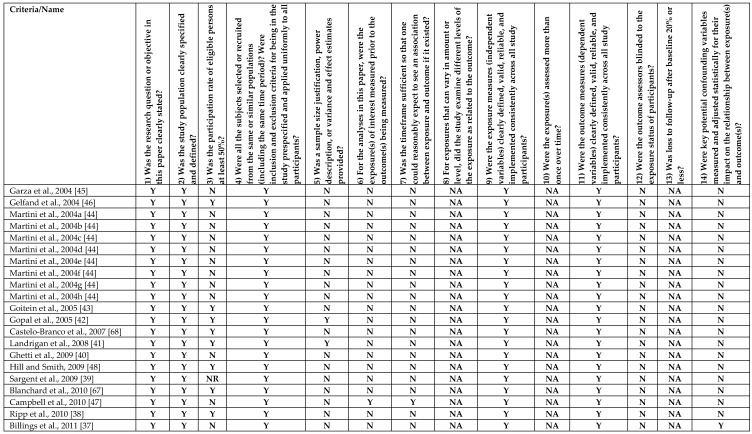

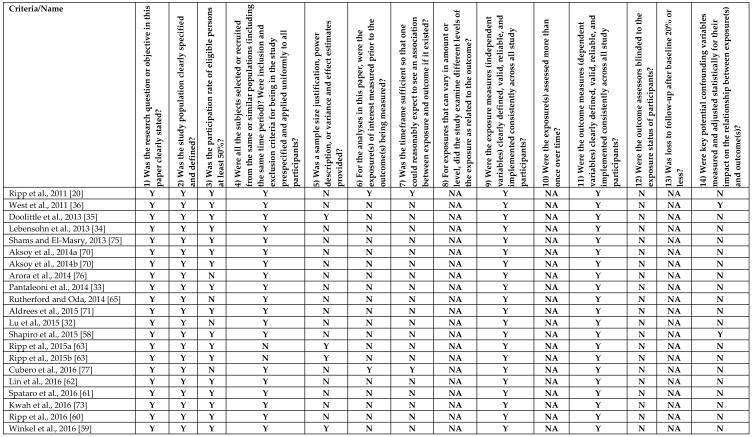

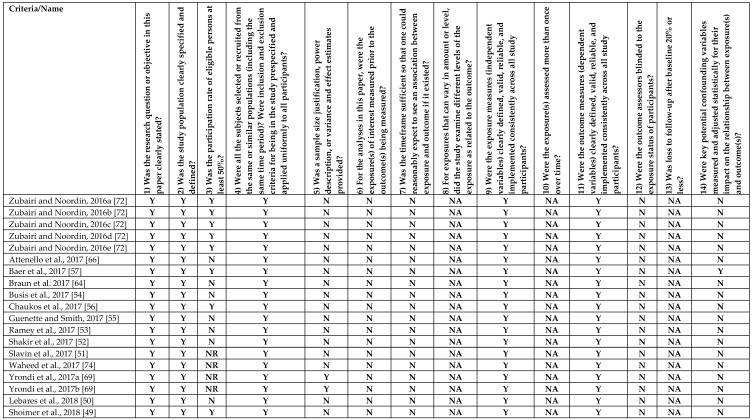
Summary of quality assessment of included studies. Y: Yes; N: No; NA: not applicable; NR: not reported.
